# Adverse life events and delinquent behavior among Kenyan adolescents: a cross-sectional study on the protective role of parental monitoring, religiosity, and self-esteem

**DOI:** 10.1186/1753-2000-8-24

**Published:** 2014-08-27

**Authors:** Caroline W Kabiru, Patricia Elung’ata, Sanyu A Mojola, Donatien Beguy

**Affiliations:** 1 African Population and Health Research Center, 2nd Floor APHRC Campus, Manga Close Off Kirawa Road, P.O. Box 10787–00100, Nairobi, Kenya; 2 Department of Sociology and Institute of Behavioral Science, University of Colorado-Boulder, 219 Ketchum Hall, 327 UCB, Boulder, CO 80309, USA

**Keywords:** Adolescents, Adverse life events, Resilience, Problem behavior theory, Kenya, Sub-Saharan Africa

## Abstract

**Background:**

Past research provides strong evidence that adverse life events heighten the risk of delinquent behavior among adolescents. Urban informal (slum) settlements in sub-Saharan Africa are marked by extreme adversity. However, the prevalence and consequences of adverse life events as well as protective factors that can mitigate the effects of exposure to these events in slum settlements is largely understudied. We examine two research questions. First, are adverse life events experienced at the individual and household level associated with a higher likelihood of delinquent behavior among adolescents living in two slums in Nairobi, Kenya? Second, are parental monitoring, religiosity, and self-esteem protective against delinquency in a context of high adversity?

**Methods:**

We used cross-sectional data from 3,064 males and females aged 12–19 years who participated in the Transitions to Adulthood Study. We examined the extent to which a composite index of adverse life events was associated with delinquent behavior (measured using a composite index derived from nine items). We also examined the direct and moderating effects of three protective factors: parental monitoring, religiosity, and self-esteem.

**Results:**

Fifty-four percent of adolescents reported at least one adverse life event, while 18% reported three or more adverse events. For both males and females, adversity was positively and significantly associated with delinquency in bivariate and multivariate models. Negative associations were observed between the protective factors and delinquency. Significant *adverse events × protective factor* interaction terms were observed for parental monitoring (females and males), religiosity (males), and self-esteem (females).

**Conclusions:**

Similar to research in high income countries, adverse life events are associated with an increased likelihood of delinquent behavior among adolescents living in urban slums in Kenya, a low-income country. However, parental monitoring, religiosity, and self-esteem may moderate the effect of adversity on delinquent behavior and pinpoint possible avenues to develop interventions to reduce delinquency in resource-poor settings in low and middle income countries.

## Background

A large body of evidence shows that adverse events in childhood and adolescence are associated with an increased likelihood of delinquent and risk behavior. For example, adverse childhood experiences, such as sexual abuse and household dysfunction, have been shown to be associated with teenage drug and alcohol use, violence perpetration, bullying, as well as early sexual intercourse [1-4]. Duke and colleagues [4] also documented a co-occurrence of adverse childhood events based on a large scale sample of adolescents aged 10–19 years in the United States (US) and showed that adolescents who had experienced multiple adverse events were more likely to report violence perpetration towards others.

Adolescents growing up in slum settings encounter a number of adverse life events, including extreme poverty, poor housing, and persistent exposure to neighborhood crime and violence, which are significantly associated with delinquency [5-9]. Multiple pathways through which adverse life events lead to delinquency and behavioral problems have been suggested in the literature. Simons and Burt [9], for example, postulate that adverse conditions, including community disadvantage and neighborhood crime, promote social schemas—a hostile, distrustful view of people, the need for immediate gratification, and a cynical view of social norms and codes of conduct—that support delinquent or criminal behavior. Gerson and Rappaport [10] also suggest that exposure to violence can lead to reactive aggression.

Although the bulk of existing studies examining associations between adversity and risk and delinquent behavior have been conducted in high-income settings in the global north, a few studies conducted in sub-Saharan Africa have documented a higher likelihood of problem behaviors among young people reporting adverse life events. One study conducted in the urban slums of Nairobi, Kenya, for example, found a strong association between self-reported coerced sexual activity and alcohol use among young people aged 12–24 years [11]. Similarly, researchers in a multi-country study conducted in Burkina Faso, Ghana, Malawi and Uganda found that adolescents reporting adverse childhood events, including physical abuse and living in a household that suffered because of a household member’s heavy drinking, were more likely to report substance use [12]. In the latter study, researchers further observed a graded association between the number of adverse events reported and the likelihood of self-reported substance use.

Empirical research on adverse childhood events has tended to focus on the risk factors for, outcomes of and potential pathways through which adverse or traumatic events in childhood may lead to various outcomes. However, living in adversity does not inevitably lead to delinquency. Many young people growing up in contexts marked by community disadvantage and high levels of violence and dysfunction are resilient and often able to overcome “the negative effects of risk exposure, cop[e] successfully with traumatic experiences and avoid the negative trajectories associated with risks.” (p. 399) [13]. Indeed, factors that are protective for youth in several settings, such as religiosity [14], may arguably take on more salience among youth at particular risk of engaging in delinquent behavior, in buffering them from their circumstances, and helping them find alternative ways of coping with adversity.

In this study, we examine the extent to which exposure to adverse life events was associated with delinquent behavior among 3,064 adolescents aged 12–19 years living in two slum settlements in Nairobi, Kenya’s capital city. Further, in order to suggest how alternative positive pathways for youth living in extreme poverty may be attained, we examine whether parental monitoring, religiosity, and self-esteem are protective in situations of adversity by assessing whether these variables moderate the association between adverse life events and delinquent behavior.

Slums or urban informal settlements, which are ubiquitous in most African cities [15], are a unique environment to examine the behavioral ramifications of exposure to adverse life events. Slum settlements are characterized by insecurity, extreme deprivation, lack of basic infrastructure, limited socio-economic and educational opportunities, and high levels of violence [15]. As noted by Ompad [16], “slum dwellers are often a particularly vulnerable group for a variety of reasons including precarious or nonexistent land tenure, lack of urban resource infrastructure, and tenuous relationships with governments and law enforcement” (p. i43).

To guide our examination of the behavioral consequences of adverse childhood events as well as potential moderators of the association between these adverse experiences and delinquency, we draw on constructs from Jessor’s Problem Behavior Theory [14,17,18]. The framework posits that behavior is influenced by protective factors and risk factors at individual or contextual levels [18]. For example, at the contextual level, protective factors, such as parental monitoring, religiosity, or perceived self-worth, promote positive (pro-social or health enhancing) behavior. Risk factors, on the other hand, increase the likelihood of risk or problem behavior. Protective factors may not only inhibit delinquency, but may also in the event of adverse events, moderate the impact of exposure to risk. We also draw on the literature on resilience, which highlights successful adaptations in the presence of risk or adversity and underscores the importance of protective factors [13,19,20].

We postulate that adolescents reporting adverse life events would report higher levels of delinquent behavior and that there would be a graded association between the reported number of adverse life events and the level of engagement in delinquent behavior. However, we also hypothesize that in line with both the Problem Behavior Theory and the concept of resilience, young people who report adverse life events, but who also report high levels of the protective factors (religiosity, parental monitoring, and self-esteem) would be less likely to engage in delinquent behavior compared with peers reporting low levels of these protective factors. Finally, we also examine whether there are gender differences in these associations.

## Methods

### Setting

Our data come from two slums in Nairobi (Korogocho and Viwandani). Korogocho is one of the oldest and most congested slum settlements in the city. Many of the residents in Korogocho have lived there for years. Viwandani, on the other hand, is situated in the city’s industrial area and is home to a youthful, relatively well-educated migrant population seeking employment in nearby industries. Both slums have limited formal health, education, and other social services [21,22] in large part because the “informal” or “squatter” nature of these settlements has long meant that these areas have been considered illegal and therefore have been marginalized by the local and national governments. As in other slums in many parts of sub-Saharan Africa [23], these slums are characterized by extreme poverty, insecurity, and crime.

### Data

We used data collected during the baseline survey of the Transitions to Adulthood Study. The study was nested in the Nairobi Urban Health and Demographic Surveillance System (NUHDSS), which has followed approximately 75,000 individuals living in more than 23,000 households in Korogocho and Viwandani since 2002. The NUHDSS collects vital health and demographic information including births, deaths and migrations occurring among residents in households within the surveillance area. These data are collected three times a year. Individuals qualify to become Health and Demographic Surveillance System (HDSS) residents through baseline enumeration, in-migration or birth [24].

Participants were randomly selected within the households in the study area using records of residents in the NUHDSS for the year 2007. Allowing for an annual attrition rate of 16% for Korogocho and 24% for Viwandani, and given the planned 3-year follow-up, a total of 5,281 young people aged 12–22 years were identified within households in the NUHDSS and targeted for recruitment. During the baseline survey 4,058 youth (50% males) aged 12–22 years were interviewed. This number reflects a 77% response rate among age-eligible young people. Overall, refusals were low (<5%) among youth whom fieldworkers were able to reach. Most of those who did not participate could not be located given the high mobility of residents in the area [25]. Participants were more likely to be from Korogocho (79% in Korogocho versus 74% in Viwandani, *p <* .05) and to be younger (16.5 years versus 16.7 years, *p <* .10) than eligible non-participants. Participants did not differ from eligible non-participants by sex. To capture participants within the adolescent age-bracket as defined by the World Health Organization [26], we restricted the analytical sample to 3,064 participants (1,566 males and 1,498 females) aged 12–19 years.

### Procedures

The baseline survey was conducted between October 2007 and June 2008. The survey collected information on, among other details, socio-demographic and behavioral characteristics, perceived parental monitoring, self-esteem, and religiosity. The questionnaire incorporated questions and measurement scales drawn from existing instruments that have been used and validated in studies conducted in various settings internationally including the Cape Area Panel Study (CAPS) [27], the National Study of Youth and Religion [28], and the Adolescent Health and Development Questionnaire [29]. The final version of the questionnaire was reviewed by an international panel of adolescent research experts. The questionnaire was also pilot-tested among adolescents living in the two slums but outside the study catchment area. The questionnaire was translated from English to Swahili, the language that was used for interviews. The original and translated versions were then reviewed by bilingual researchers and interviewers to ensure comparability.

The interviews were conducted in Kiswahili by male and female interviewers, many of whom had previous experience working in Korogocho and Viwandani. Prior to fieldwork, interviewers participated in a 5-day training workshop that included sessions on the objectives of the Transitions to Adulthood study, the study tools, the roles of interviewers, and ethical practices in research. The training also included mock interviews and pilot interviews with a group of youth living in the two slum settlements but outside the study area. Interviews were conducted in the participants’ homes or other private settings and lasted about 1 hour, on average. Due to the sensitive nature of questions about adverse experiences, these questions were only asked if there was no one over three years of age within listening distance.

Approval to conduct the study was obtained from the Kenya Medical Research Institute’s Ethical Review Committee. All participants provided written or oral consent. For participants aged 12–17, parental consent was obtained prior to seeking assent from the adolescent.

### Measures

#### Outcome variable

Similar to other studies that have assessed delinquency [30,31], the primary outcome variable, *delinquent behavior,* was assessed using a composite index derived from standardized values of seven items that measured the frequency (0 = *never*; 1 = *once*; 2 = *more than once*) with which youth engaged in the following behaviors in the 4 months preceding the survey: staying away from home for at least one night without parental permission; starting a fight with peers; taking or trying to take something belonging to someone else without their knowledge; carrying a knife, gun, or other weapon; hitting or threatening to hit a peer or adult; delivering or selling drugs; and delivering or selling alcohol. In line with previous literature that considers early sex and multiple sexual partnerships as problem behaviors [32], well as literature showing a strong association between sexual behavior and delinquency [30], we also included two items assessing whether the participant had engaged in sexual intercourse by age 15 and whether the participant had ever had multiple sexual partners. Internal consistency of scores on the delinquency scale was assessed using Cronbach’s alpha [33]. The Cronbach’s alpha value for item scores on the delinquency scale was 0.73. A higher score on the scale indicates more frequent involvement in delinquent behavior.

#### Explanatory variables

The primary explanatory variable, *exposure to adverse life events,* was defined as participants’ self-reported experience of potentially stressful and undesirable events experienced personally or at family/household level [34]. This variable was assessed using 10 items (Table 1) that assessed adverse events experienced personally (four items, e.g., Sometimes people do things to us we do not want. Has anyone ever touched you in an unwanted sexual way, such as kissing, grabbing or fondling?), and at family or household level (six items, e.g., in the last month, has your family/household ever not had enough food to feed everyone?). We computed a composite adverse events score using standardized values of the 10 items. In addition, we created a categorical variable with four levels (no adverse life events reported, one event, two events, and three or more) showing the total number of adverse events reported by each participant.

**Table 1:** Proportion of participants reporting adverse life events and t-test statistics for differences in delinquency between those who have experienced an adverse event and those who have not, by sex

	**Male n = 1,566**		**Female n = 1,498**		**Total n = 3,064**
	**%**	***t *** **(*df)***	**%**	***t *** **( *df *)**	**%**
In the last month, family did not have enough food^a, b^	29.4	-7.9 (1530)	27.4	-8.1 (1456)	28.5
In the last three months, family suffered because parent(s) were out of a job^a, b^	37.7	-9.7 (1487)	38.1	-9.9 (1394)	37.9
Ever lived with a problem drinker or alcoholic^a, b^	11.8	-10.3 (1506)	12.2	-9.4 (1434)	12.0
Parents ever divorced or separated^a, b, c^	11.9	-10.1 (1529)	9.6	-9.1 (1453)	10.8
Ever lost your home because of a disaster?^a^	11.2	-5.9 (1466)	12.5	-4.8 (1399)	11.8
Ever witnessed mother/mother figure being beaten^a, b^	10.9	-2.9 (1530)	12.4	-0.2 (1455)	11.7
Ever kicked out of the home by a parent/guardian?^a, b^	4.5	-11.0 (1513)	3.2	-7.3 (1442)	3.9
Ever had a parent or adult living in same home inflict injury^a, b^	10.5	-7.1 (1501)	9.8	-9.4 (1439)	10.1
Ever been touched in unwanted sexual way^a, b, c^	1.3	-2.2 (1531)	5.6	-10.2 (1456)	3.4
Ever been physically forced to have sexual intercourse^a, b, c^	0.5	-3.1 (1530)	2.7	-11.5 (1453)	1.6
Number of adverse experiences (*χ*^*2*^ (*3,* N=3,064) = 0.12)					
None	45.8	-	45.4	-	45.6
One	17.2	-	17.3	-	17.2
Two	18.1	-	18.5	-	18.3
Three or more	18.9	-	18.8	-	18.9

^a, b^p < .05 for differences in delinquency between males (a) and females (b) who have experienced the adverse event and those who have not based on t-tests.

^c^p < .05 for differences between males and females based on chi-square tests.

Three types of protective factors were assessed: parental monitoring, religiosity, and self-esteem. Parental monitoring—perceptions of parents’ knowledge and supervision of participants’ friends, whereabouts and activities [35]—was measured using nine items (e.g., How much would you say your parents/guardians really know about where you spend time in the evenings on weekdays? Response options: they never know, sometimes know, always know) (Cronbach’s α = .97). Religiosity—the role of religion in participants’ lives [36]—was a composite measure created using five items that assessed the frequency of participation in religious services (i.e., how many times have you gone to religious services during the past one month? Response options: never, 1 time, 2–3 times, 4 times, more than 4 times) and the importance of relying on religious teachings and beliefs, believing in God, and prayer in one’s life (e.g., how important is it to you to be able to rely on religious teachings when you have a problem? Response options: not important, somewhat important, important, very important) (Cronbach’s α = .93). Self-esteem—defined as participants’ overall sense of self-worth and adequacy [37]—was measured using five items assessing the adolescent’s ability to get along with others, living up to expectations, ability to do well in school, self-rated attractiveness, and self-satisfaction (e.g., On the whole, how satisfied are you with yourself? Response options: very satisfied, pretty satisfied, not too satisfied, not satisfied at all) (Cronbach’s α = .61).

### Analyses

Descriptive statistics of the participants’ social and demographic characteristics, engagement in delinquent behavior, as well as experience of adverse life events were computed (Table 1). Bivariate analyses (chi-squares, Pearson’s correlations, ANOVA, and t-tests) were conducted to examine gender differences in the study variables (Tables 1 and 2) and to examine pairwise correlations between the primary explanatory variables and delinquency (Table 3). We then run multivariate linear regression models to examine the association between the explanatory variables and the delinquency measure controlling for socio-demographic variables (age, study site, and schooling status) (Table 4). We ran two models. The first included the primary explanatory variables and socio-demographic controls. The second model added interaction terms between the protective factors and the number of adverse events. Since some households had more than one adolescent, the models were adjusted for cluster effects. To check that the assumptions for linear regression analyses were not violated, we tested the normality of the regression residuals as suggested by Li and colleagues [38]. All analyses were conducted separately for males and females, using Stata 12.0 [39]. For bivariate and multivariate analyses, a p-value of less than 0.05 was considered statistically significant. Fewer than 5% of participants had missing information on variables included in the multivariate models thus no imputation was performed.

**Table 2:** Descriptive statistics of adolescents by sex

	**Male n = 1,566**	**Female n = 1,498**	**Total N = 3,064**
	**%**	**%**	**%**
Currently attending school (*χ*^2^ (1, N=3,063) = 15.39)*			
Yes	74.3	67.9	71.2
No	25.7	32.1	28.8
Residence (*χ*^2^ (1, N=3,060) = 0.32)			
Korogocho	50.3	51.3	50.8
Viwandani	49.7	48.7	49.2
Religious affiliation (*χ*^2^ (6, N=3,061) = 53.38)*			
Catholic	27.7	27.5	27.6
Protestant	21.4	22.1	21.7
Pentecostal	19.2	26.1	22.6
Other Christian	2.9	2.9	2.9
Muslim	13.4	11.9	12.6
No religion	10.7	4.7	7.7
Other	4.7	4.8	4.8
Mean delinquency (SD) (*t* = 9.34; *df* = 3,062)*^, a^	0.10 (0.66)	-0.09 (0.43)	0.00 (0.57)
Raw delinquency (*t* = 10.25; *df* = 3,062)*	0.25 (0.32)	0.15 (0.23)	0.20 (0.28)
Mean parental monitoring (SD) (*t* = -5.68; *df* = 3,014)* ^a^	-0.09 (0.87)	0.09 (0.89)	0.00 (0.88)
Raw parental monitoring (*t* = -5.69; *df* = 3,014)*	2.15 (0.83)	2.33 (0.84)	2.24 (0.84)
Mean religiosity (SD) (*t* = -7.53; *df* = 3,056)*^, a^	-0.12 (1.01)	0.12 (0.73)	0.00 (0.89)
Raw religiosity (*t* = -7.58; *df* = 3,056)*	3.08 (1.17)	3.36 (0.85)	3.21 (1.03)
Mean esteem (SD) (*t* = -0.52; *df* = 3,061)^a^	-0.01 (0.64)	0.01 (0.61)	0.00 (0.63)
Raw esteem (*t* = -0.56;* df* = 3,061)	-1.55 (0.44)	-1.54 (0.41)	-1.55 (0.42)

*p < 0.05 for sex differences based on chi-square tests (categorical variables) and t-tests (continuous variables).

^a^Indices generated from standardized (mean equal to zero and standard deviation equal to one) values of individual items all scored in the positive direction. Number of items in scales: delinquency (9 items); religiosity (5 items); parental monitoring (9 items); self-esteem (5 items).

**Table 3:** Pearson’s correlation coefficients between delinquency and the primary explanatory variables, by sex

	**Delinquency**	**Adverse life events**	**Parental monitoring**	**Religiosity**	**Self-esteem**
*Males*					
Delinquency	1				
Adverse life events	0.37*	1			
Parental monitoring	-0.28*	-0.14*	1		
Religiosity	-0.22*	-0.14*	0.23*	1	
Self-esteem	-0.24*	-0.28*	0.23*	0.24*	1
*Females*					
Delinquency	1				
Adverse life events	0.41*	1			
Parental monitoring	-0.25*	-0.11*	1		
Religiosity	-0.16*	-0.15*	0.17*	1	
Self-esteem	-0.22*	-0.20*	0.13*	0.14*	1

*p < 0.05.

**Table 4:** Linear regression of delinquency on number of adverse events, socio-demographics, and protective factors by sex

	**Male**		**Female**	
	***Model 1 β *** **(SE)**	***Model 2 β *** **(SE)**	***Model 3 β *** **(SE)**	***Model 4 β *** **(SE)**
Adverse events (AE)	0.40(0.04)***	0.30(0.04)***	0.27(0.03)***	0.22(0.02)***
Parental monitoring	-0.11(0.02)***	-0.11(0.02)***	-0.05(0.01)***	-0.05(0.01)***
Religiosity	-0.07(0.02)***	-0.06(0.02)***	-0.04(0.02)*	-0.04(0.01)**
Self-esteem	-0.09(0.03)***	-0.08(0.03)**	-0.08(0.02)***	-0.07(0.02)***
15-19 years (ref. 12–14 years)	-0.04 (0.03)	-0.01 (0.03)	0.00 (0.02)	0.00 (0.02)
Out of school (ref. in school)	0.16(0.05)***	0.15(0.05)**	0.11(0.03)***	0.11(0.03)***
Viwandani (ref. Korogocho)	-0.18(0.03)***	-0.17(0.03)***	-0.11(0.02)***	-0.11(0.02)***
AE × parental monitoring		-0.11(0.05)*		-0.08(0.03)**
AE × religiosity		-0.15(0.04)***		0.00 (0.03)
AE × self-esteem		-0.05 (0.06)		-0.13(0.05)*
Constant	0.16(0.03)***	0.12(0.03)***	-0.06(0.02)***	-0.08(0.02)***
Observations (*N*)	1498	1498	1436	1436
Adjusted R-squared (*R*^*2*^)	0.23	0.26	0.25	0.27

Ref = reference category.

*p < 0.05, **p < 0.01, ***p < 0.001.

## Results

### Descriptive analysis

Table 2 summarizes the socio-demographic characteristics of the 3,064 participants in the analytic sample. Overall, 71% of participants were attending school at the time of the survey. A significantly higher proportion of males (74%) than females (68%) were currently in school. The number of participants was equally split across the study sites. About three quarters of the participants were Christians. Males were significantly more likely than females to report delinquent behavior. Females reported higher levels of religiosity and parental monitoring. There were no significant sex differences on the self-esteem scale. For both males and females, delinquency was negatively correlated with religiosity, parental monitoring, and self-esteem (Table 3). All correlations were significant at the .05 level.

Overall, 54% of participants reported that they had experienced at least one adverse life event (Table 1). The most frequently reported adverse events were that the family had suffered in the three months preceding the survey because a parent or both parents were out of a job (38%), and that the household suffered food insecurity in the month preceding the survey (29%). Less than 5% of adolescents reported that they had ever been kicked out of home by a parent or guardian (4%), been touched in an unwanted sexual way (3%), or been physically forced into having sexual intercourse (2%). However, females were more likely than males to report unwanted sexual touching and coerced sexual intercourse. Males, on the other hand, were more likely to report that their parents had divorced or separated.

Individual adverse events were significantly associated with delinquency at bivariate level (with the exception of ever losing home because of a disaster among females). In each case, adolescents who had experienced an adverse event had higher scores on the delinquency scale than those who had not experienced the event (Table 1). The cumulative number of adverse events experienced was also significantly associated with delinquent behavior at the bivariate level (males F (3, 1562) = 51.0, p < 0.05; females F (3, 1494) = 59.8, p < 0.05). Pairwise comparisons showed that for both males and females, participants reporting at least one adverse life event had higher delinquency scores than those reporting no adverse event and that there was a positive association between the number of events reported and delinquency.

### Multivariate analyses

**Figure 1:**
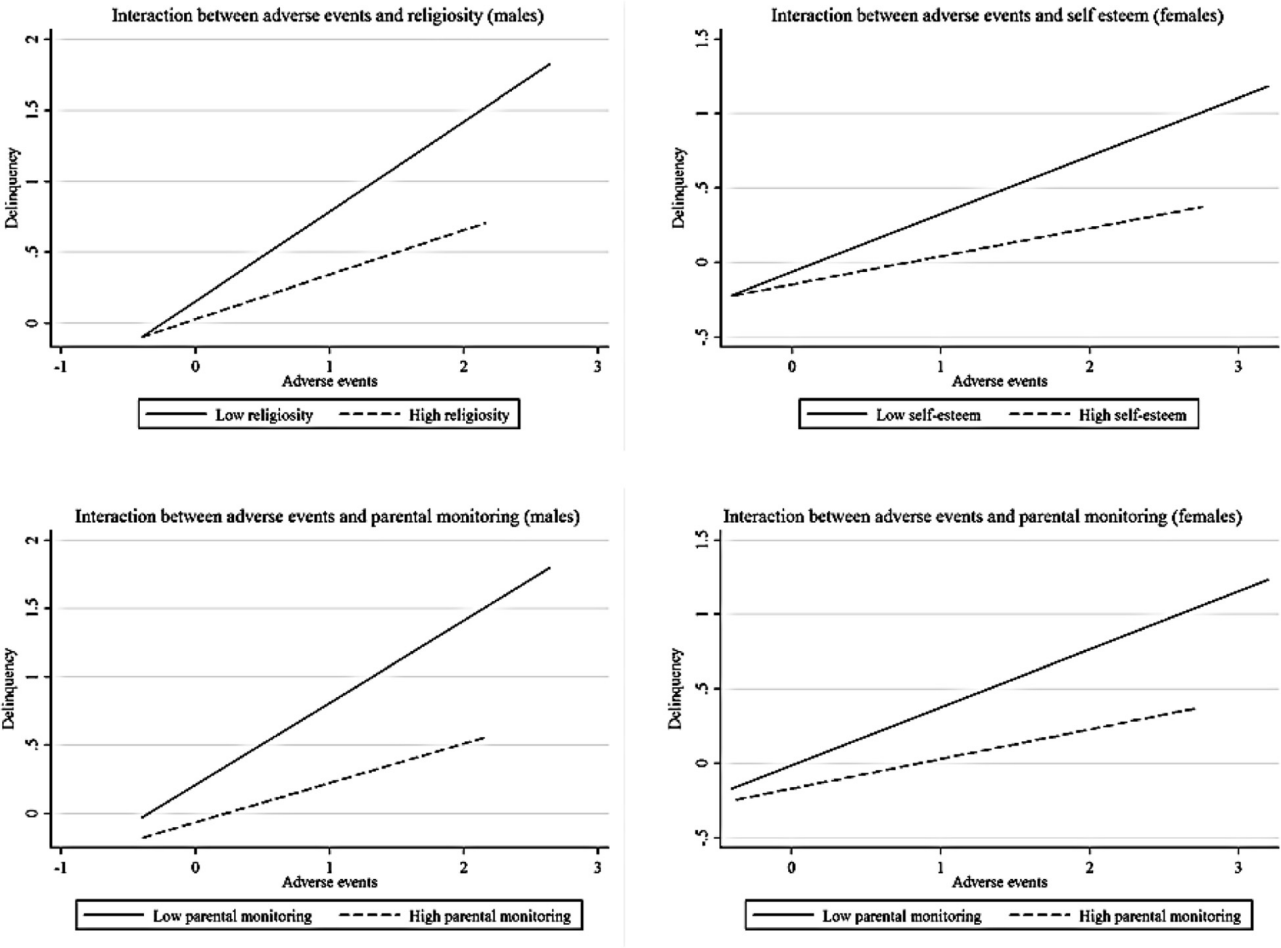
Illustration of the moderator effects of protective factors on the relationship between delinquency and number of adverse events among males and females 12–19 years.

In the multivariate analysis (Table 4), the adverse events variable was positively and significantly associated with delinquent behavior after controlling for protective factors and socio-demographic variables for both males (model 1) and females (model 3). The coefficients remained significant– albeit attenuated–after including interaction terms between the adverse events variable and the protective factors (models 2 and 4). All three protective factors were negatively and significantly associated with delinquency in the multivariate regression models for males (model 1) and females (model 3). The regression coefficients remained significant when interactions between the protective factors and the number of adverse life events experienced were included in the model (models 2 and 4). Among the control variables, schooling status, and area of residence were significantly associated with delinquent behavior. Specifically, being out of school and living in Korogocho were associated with higher delinquency scores (models 1 and 3).For both males and females, inclusion of the interaction effects resulted in a small increase in the proportion of variance explained (3% and 2%, respectively). Among males, the interactions between the adverse events and religiosity variables as well as between the adverse events and parental monitoring variables were significant (model 2). For females, the interactions between the adverse events variable and the parental monitoring and self-esteem variables were statistically significant (model 4). To illustrate these interaction effects, we created categorical variables showing high and low levels of protective factors (the group labeled as ‘high’ comprises the approximately 50% of participants who had the highest scores on the respective index, and vice versa) for each category of number of adverse life events and plotted the mean delinquency by adverse events for each new category. Figure 1 shows that for males, the strength of the association between adverse life events and delinquency was attenuated by high levels of religiosity and parental monitoring. Among females, the strength of the association between adverse life events and delinquency was attenuated by high levels of parental monitoring and self-esteem.

## Discussion

An extensive body of research exists on the links between adverse or traumatic events in childhood and behavioral and psychosocial outcomes later in life. However, much of this research is based on studies conducted in the global north. Further, existing literature tends to lay emphasis on the risks associated with exposure to adversity with less attention focused on protective factors that may buffer individuals from the negative outcomes stemming from adverse life experiences. In this study, we advance the literature in two ways. First, we examine the extent to which experience of adverse life events was associated with delinquent behavior among adolescents aged 12–19 years who live in two slum settlements in Nairobi, Kenya. Second, we examine whether three protective factors, which are indicators of different domains of young people’s lives; individual level (self-esteem, religiosity) and household level (parental monitoring), moderated the association between adverse life events and delinquent behavior.

### Adversity and delinquency

We found that more than half of the adolescents lived in households characterized by either food insecurity or recent parental unemployment, and almost a fifth had dealt with multiple adverse events. Consistent with previous studies [4,34], we observed a strong association between the number of adverse life events and delinquency. However, the findings from our study also demonstrate the role that protective factors may play in moderating the association between adverse life events and delinquent behavior, and highlight important gender differences.

### Parental monitoring

For both males and females, we observed a significant interaction between parental monitoring and the number of adverse life events, with adolescents with high levels of parental monitoring also reporting lower levels of delinquency even at high levels of adversity. Parental closeness is an important support and buffer for children living in contexts characterized by high levels of adversity because the close ties between the parent and child allow for greater self-expression and enables parents to provide better care for their children [40]. In addition, close parental monitoring may help young people adjust positively when exposed to external stressors [40]. Our study adds international breadth to the large literature on the association between parental involvement and delinquency, especially among youth in resource-poor contexts [5-8] where literature from youth living in extreme poverty in low and middle income countries is limited. The positive influence of parents found here suggests their useful focus in youth interventions conducted in resource-poor settings. Indeed existing studies suggest that targeting parents may be an important approach to reduce both the incidence of and outcomes of adverse life experiences. One study conducted in the US [41], for example, provides evidence that a population-based parenting intervention can reduce child maltreatment rates. Further investigations of whether similar programs can be implemented in resource-poor settings in low and middle countries and achieve similar results would be useful in informing policies and programs to enhance positive youth development in contexts marked by pervasive adversity.

### Religiosity

Similar to other studies in the global north [36,42,43], we found that young people reporting high religiosity had lower delinquency scores even in situations of high adversity. Overall, it is likely that religious teachings enable young people to cope with possibly traumatic events in ‘positive’ ways by proscribing delinquent behavior, enhancing self-control, and shaping pro-social attitudes and beliefs while at the same time providing support to those who have experienced adverse events [44]. In Kenya, over 85% of people say religion is very important to their lives [45]. Previous research in Kenya [46-49] has highlighted the importance of religion in enabling youth to cope with difficult life situations and adversity. Our findings further bridge the gap between the declared importance of religion in the lives of Kenyans and its significance in shaping youth outcomes in adverse situations and contexts. Religion may influence youth not only through activities such as regular church attendance, but also through attending youth groups, and engaging in religious coping activities such as personal prayer. As poor youth transition to adulthood and are persistently exposed to adverse and sometimes criminogenic settings [9,50], religiosity may therefore not only shape their self-perceptions, but also the kinds of moral rules and regulations they take on from their environment.

### Self-esteem

There has been a debate in the literature in the global north as to whether self-esteem has an effect on youth delinquency, with studies finding no effect, mixed effects or positive effects [37]. In this study, we observed a direct association between self-esteem and delinquency for males and females with higher levels of self-esteem associated with a lower likelihood of reporting delinquent behavior. A study conducted in Rwanda by Betancourt et al. [40] among children affected by HIV/AIDS, their caregivers, and other key informants also identified self-esteem as a key protective factor in situations of adversity. In the Rwandan study, children with high self-esteem were viewed as being more courageous, better able to overcome difficulty, and able to believe in a better future despite hardships experienced in the present. These findings suggest that interventions that enhance positive self-perceptions among adolescents who are prone to adversity may improve behavioral outcomes.

### Gender differences

While parental monitoring was significant for both males and females, we found significant gender differences in religiosity and self-esteem in ameliorating delinquency among youth. The gender differences in the role of religiosity in this study are noteworthy. While a recent study in the US found a gender invariant protective effect of religion on delinquency [51], in this study, the buffering effect of religiosity in this setting was only significant for males. It is plausible that because females reported higher levels of religiosity, lower levels of variability in religiosity among them may mask the potentially protective role that religiosity plays. Further, the protective features of religion for boys may include the ability to engage in social institutions, such as church and religious youth groups, that provide sanctions for deviant behavior, models for conventional behavior and positive youth development [13,14], and provide concrete alternatives to gangs and other groups that are commonly associated with male delinquency.

We also found important gender differences in the role of self-esteem in buffering the association between adverse life events and delinquency. Self-esteem was only significantly protective for females. Our findings are in line with previous research in the US which found significant associations between low self-esteem, early sex, and risky sexual partnerships among adolescent females [52]. Previous research shows that males score slightly higher on measures of global self-esteem scales with the largest effect of age observed in late adolescence [53]. It is therefore plausible that males in this study may have a lower level of variability in self-esteem making it difficult to observe the moderating effect of self-esteem. Although we were unable to assess whether high self-esteem precedes the adverse events, we posit that young females with high levels of self-esteem have more positive self-perceptions and are able to constructively cope with stress in contexts of adversity.

Overall, our findings on gender differences in the buffering effects of religiosity for males and self-esteem for females, suggest potentially gendered pathways and mechanisms through which youth cope with adverse situations and are protected or buffered from delinquency. One reason might be linked to the gendered types of delinquency in which adolescents engage, with male delinquency linked to deviant peer groups, thus suggesting why alternative positive groups and institutions may be protective; while female delinquency or problem behavior (particularly with respect to early sexual behavior) may be linked to low self-esteem and self-image, suggesting why higher self-esteem is protective. More research is needed to examine whether these findings and potential mechanisms hold in other settings.

### Limitations

Our study should be interpreted in light of several limitations. First, this study examines a limited range of measures on delinquency and possible factors that may affect delinquency. Previous studies conducted in other settings [4] have shown that adverse childhood events are associated with both interpersonal violence perpetration and self-directed violence. Given the dearth of literature on delinquency among youth populations living in resource-poor settings in low and middle income countries, further studies that examine the prevalence and a wider range of precursors of delinquency in these settings are warranted. Second, our data preclude causal interpretations. Future research should examine whether these associations persist over time and are predictive of future delinquency. Third, this study is based on self-reported data on sensitive behaviors as well as experiences, and may be subject to self-report bias despite our efforts to assure participants that all data would be confidential. These limitations notwithstanding, the study confirms findings from other studies, largely in the global north, that demonstrate a strong link between adversity and delinquency. The study also illuminates key protective factors that may attenuate the risk for delinquency among adolescents living in challenging urban contexts in sub-Saharan Africa.

## Conclusions

Adolescents in urban slums in sub-Saharan Africa live in contexts marked by significant adversity. Similar to previous research, our results show that adverse life events are associated with an increased likelihood of risk behavior among adolescents. However, we find that parental monitoring, high religiosity, and high self-esteem may moderate the effect of adversity on risk behavior. Our findings highlight the potential benefits that interventions that nurture supportive parent–child relationships and that enhance the emotional health of adolescents may have in enabling adolescents successfully cope with adversity. In addition, our findings underscore the role that religious organizations may play in enhancing positive youth development among adolescents living in resource-poor contexts.

## Competing interests

The authors declare that they have no competing interests.

## Author contributions

CWK conceptualized the manuscript idea, reviewed literature, and prepared the first draft of the manuscript. PE made substantive contributions to the conceptualization of the manuscript, supported the literature review, and performed the statistical analysis. SM made substantive contributions to the conceptualization of the study and manuscript preparation. DB made substantive contributions to the conceptualization of the manuscript and supported the statistical analysis. All authors critically reviewed the manuscript. All authors are aware that the manuscript is being submitted to the journal. All authors read and approved the final manuscript.
